# Supplementary material for: BuDDI: Bulk Deconvolution with Domain Invariance to predict cell-type-specific perturbations from bulk
Source: PLoS Comput Biol. 2025 Jan 17;21(1):e1012742. doi: 10.1371/journal.pcbi.1012742 (PMC11790236; doi:10.1371/journal.pcbi.1012742)
Supplement: S1 Fig — Panel a depicts that average F1 score of each latent space to predict each source of variation. Midpoint coloration is the average across all observed F1 scores. Panel b compares the performance of BuDDI, CIBERSORTx, and BayesPrism, in estimating the cell type proportions. Panel c depicts each of BuDDI’s latent spaces, colored by source of variation. Panel d depicts the Pearson correlation of the simulated perturbation expression, stratified by expression level. (PDF) [file pcbi.1012742.s001.pdf]

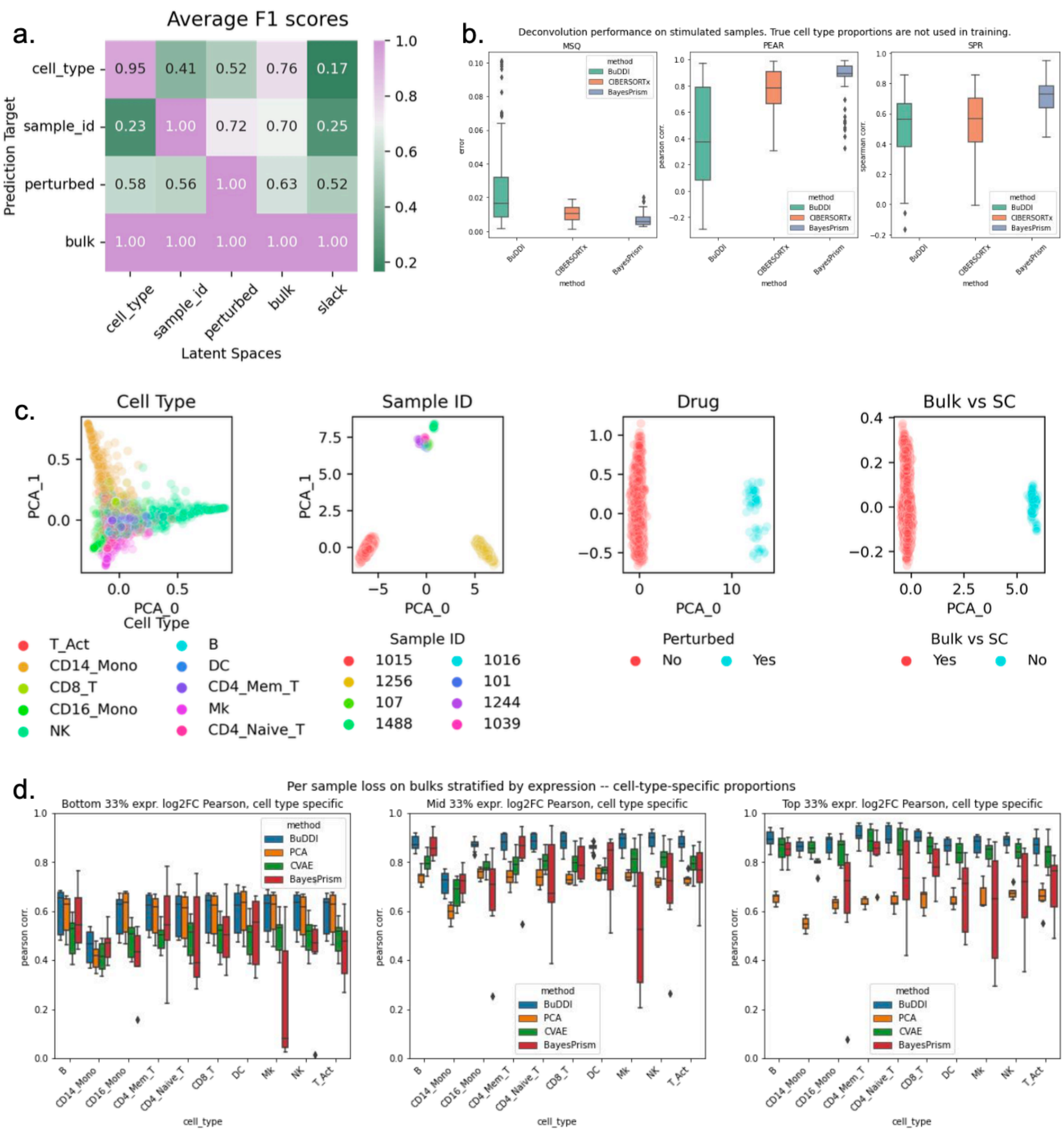

Supp Figure 1. Latent space analysis of BuDDI on Kang et al.[1] data set with an experimental design where bulk samples are correlated with the sample IDs and perturbation status. **Panel a** depicts that average F1 score of each latent space to predict each source of variation. Midpoint coloration is the average across all observed F1 scores. **Panel b** compares the performance of BuDDI, CIBERSORTx, and BayesPrism, in estimating the cell type proportions. **Panel c** depicts each of BuDDI's latent spaces, colored by source of variation. **Panel d** depicts the Pearson correlation of the simulated perturbation expression, stratified by expression level.

Reference

1. Kang HM, Subramaniam M, Targ S, Nguyen M, Maliskova L, McCarthy E, et al. Multiplexed droplet single-cell RNA-sequencing using natural genetic variation. Nat Biotechnol. 2018;36: 89–94.
