# Supplementary material for: BuDDI: Bulk Deconvolution with Domain Invariance to predict cell-type-specific perturbations from bulk
Source: PLoS Comput Biol. 2025 Jan 17;21(1):e1012742. doi: 10.1371/journal.pcbi.1012742 (PMC11790236; doi:10.1371/journal.pcbi.1012742)
Supplement: S2 Fig — Panel a Schematic of the experimental design. 15 tests were used to evaluate each model’s performance on sci-Plex3 data. Each test corresponds to a single drug and cell line combination. More specifically, each perturbed pseudobulk consists of a single cell line perturbed by a single drug, all other cell lines are unperturbed. Panel b Performance evaluation of each model on 15 sci-Plex3 tests, corresponding to specific drug and cell line combinations. True positives were the top differentially expressed genes per cell line, measured using area under the precision-recall curve (AUPRC) for the top 10 and 50 differentially expressed genes. False-positive rates were assessed by identifying genes falsely classified as differentially expressed between non-perturbed cell lines, using a Bonferroni-corrected p-value <0.05. BuDDI, PCA, and CVAE were independently trained three times, while BayesPrism used subsampled perturbed and non-perturbed data. The syringe icon was obtained from openclipart [42]. (PDF) [file pcbi.1012742.s002.pdf]

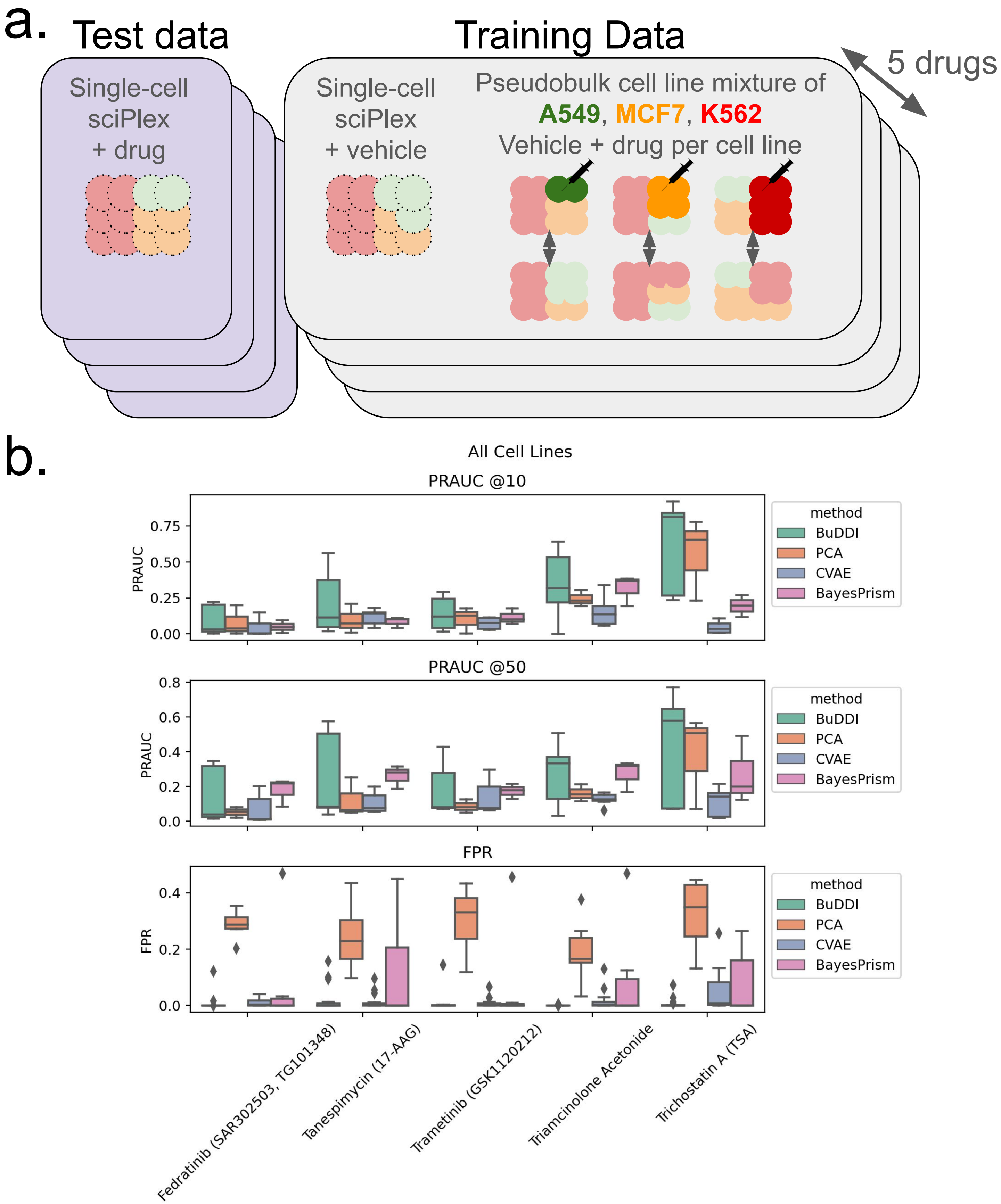

Supp Figure 2. Estimating cell line specific perturbation effects in sci-Plex3 data. **Panel a** Schematic of the experimental design. 15 tests were used to evaluate each model's performance on sci-Plex3 data. Each test corresponds to a single drug and cell line combination. More specifically, each perturbed pseudobulk consists of a single cell line perturbed by a single drug, all other cell lines are unperturbed. **Panel b** Performance evaluation of each model on 15 sci-Plex3 tests, corresponding to specific drug and cell line combinations. True positives were the top differentially expressed genes per cell line, measured using area under the precision-recall curve (AUPRC) for the top 10 and 50 differentially expressed genes. False-positive rates were assessed by identifying genes falsely classified as differentially expressed between non-perturbed cell lines, using a Bonferroni-corrected p-value < 0.05. BuDDI, PCA, and CVAE were independently trained three times, while BayesPrism used subsampled perturbed and non-perturbed data. The syringe icon was obtained from openclipart[42].
