# Supplementary material for: BuDDI: Bulk Deconvolution with Domain Invariance to predict cell-type-specific perturbations from bulk
Source: PLoS Comput Biol. 2025 Jan 17;21(1):e1012742. doi: 10.1371/journal.pcbi.1012742 (PMC11790236; doi:10.1371/journal.pcbi.1012742)
Supplement: S3 Fig — Each column is a latent space and each row is colored by a source of variation. The second row is colored by sample ID, but due to the number of bulk samples, we omit the sample ID legend. (PDF) [file pcbi.1012742.s003.pdf]

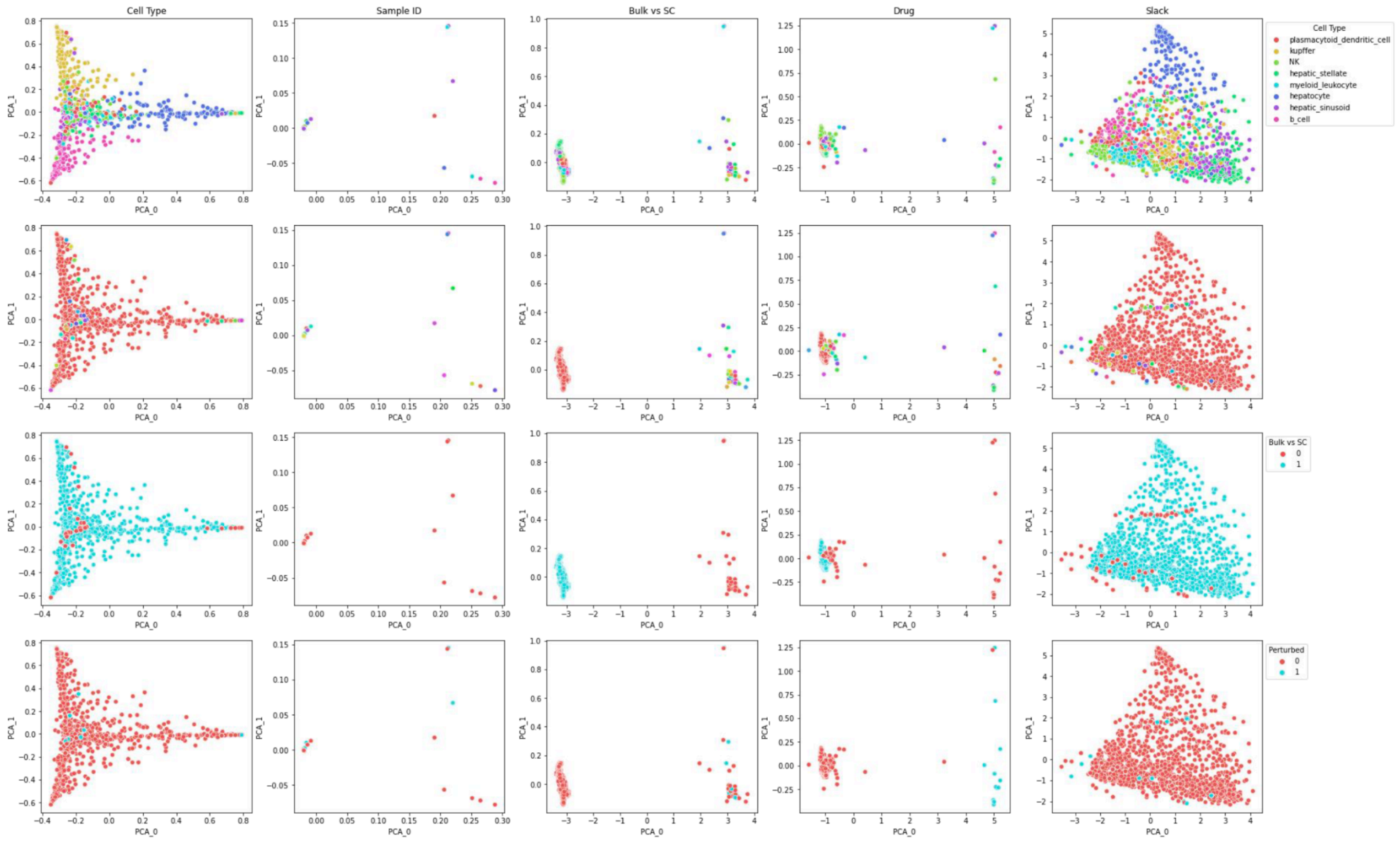

Supp Figure 3. Latent space analysis of BuDDI on Tabula Muris Senis dataset. Each column is a latent space and each row is colored by a source of variation. The second row is colored by sample ID, but due to the number of bulk samples, we omit the sample ID legend.
