# Supplementary material for: BuDDI: Bulk Deconvolution with Domain Invariance to predict cell-type-specific perturbations from bulk
Source: PLoS Comput Biol. 2025 Jan 17;21(1):e1012742. doi: 10.1371/journal.pcbi.1012742 (PMC11790236; doi:10.1371/journal.pcbi.1012742)
Supplement: S4 Fig — Top row uses the differential expressed genes form an independent single-nucleus experiment [53] as the ground truth, bottom row uses the union of the single-nucleus and our calculated single-cell results from Tabula Muris Senis [44,45] as the ground truth. (PDF) [file pcbi.1012742.s004.pdf]

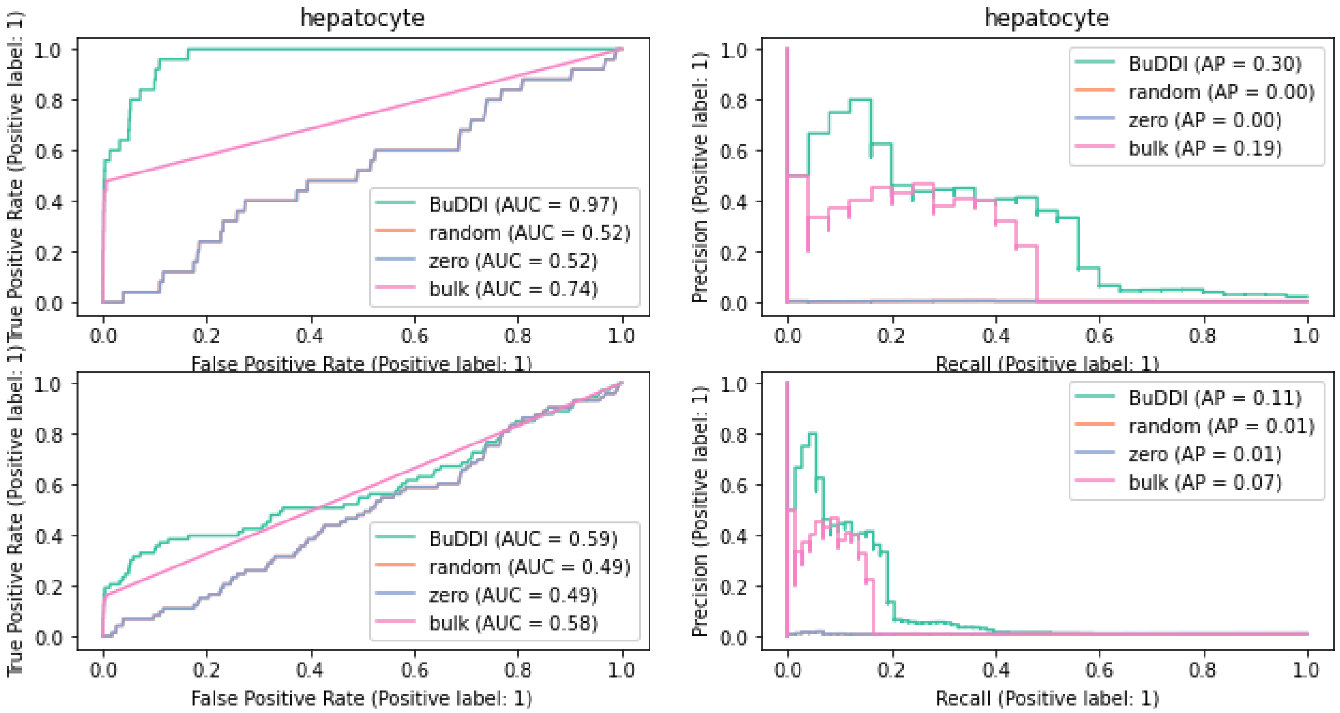

Supp Figure 4. ROC and PR curves for predicting differentially expressed genes between sexes in hepatocytes using BuDDI. Top row uses the differential expressed genes form an independent single-nucleus experiment[1] as the ground truth, bottom row uses the union of the single-nucleus and our calculated single-cell results from Tabula Muris Senis[2,3] as the ground truth.

Reference

1. Goldfarb CN, Karri K, Pyatkov M, Waxman DJ. Interplay Between GH-regulated, Sex-biased Liver Transcriptome and Hepatic Zonation Revealed by Single-Nucleus RNA Sequencing. Endocrinology. 2022;163. doi:10.1210/endocr/bqac059
2. Tabula Muris Consortium. A single-cell transcriptomic atlas characterizes ageing tissues in the mouse. Nature. 2020;583: 590–595.
3. Schaum N, Lehallier B, Hahn O, Pálovics R, Hosseinzadeh S, Lee SE, et al. Ageing hallmarks exhibit organ-specific temporal signatures. Nature. 2020;583: 596–602.
