# Supplementary material for: BuDDI: Bulk Deconvolution with Domain Invariance to predict cell-type-specific perturbations from bulk
Source: PLoS Comput Biol. 2025 Jan 17;21(1):e1012742. doi: 10.1371/journal.pcbi.1012742 (PMC11790236; doi:10.1371/journal.pcbi.1012742)
Supplement: S5 Fig — The red box highlights the true or estimated cell type proportions used in BuDDI. (PDF) [file pcbi.1012742.s005.pdf]

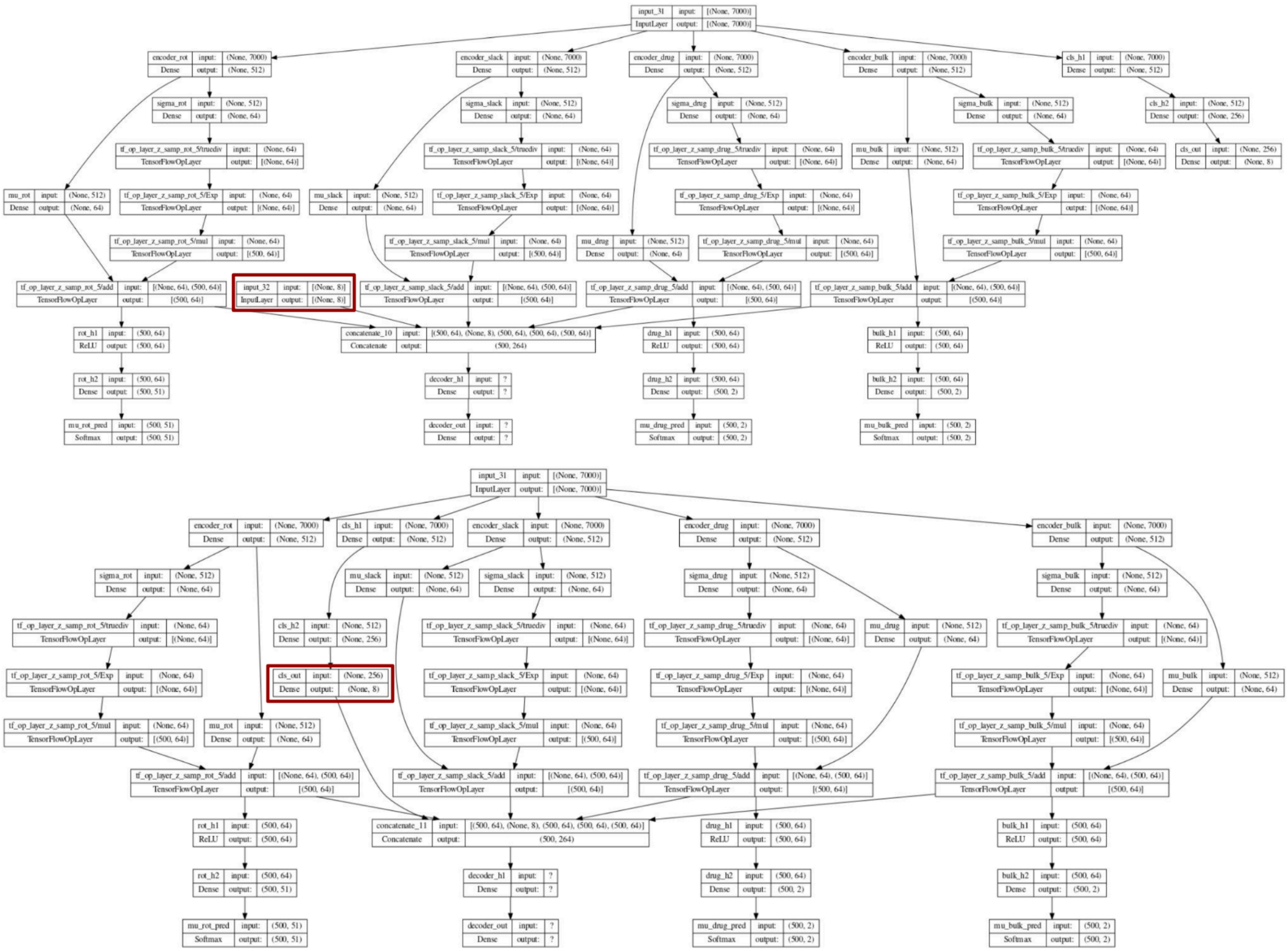

Supp Figure 5. BuDDI model overview for the supervised (top) and unsupervised (bottom) models. The red box highlights the true or estimated cell type proportions used in BuDDI.
