# Supplementary material for: BuDDI: Bulk Deconvolution with Domain Invariance to predict cell-type-specific perturbations from bulk
Source: PLoS Comput Biol. 2025 Jan 17;21(1):e1012742. doi: 10.1371/journal.pcbi.1012742 (PMC11790236; doi:10.1371/journal.pcbi.1012742)
Supplement: S6 Fig — Our generated data shows independence between, each source of variation, including cell type proportion. (PDF) [file pcbi.1012742.s006.pdf]

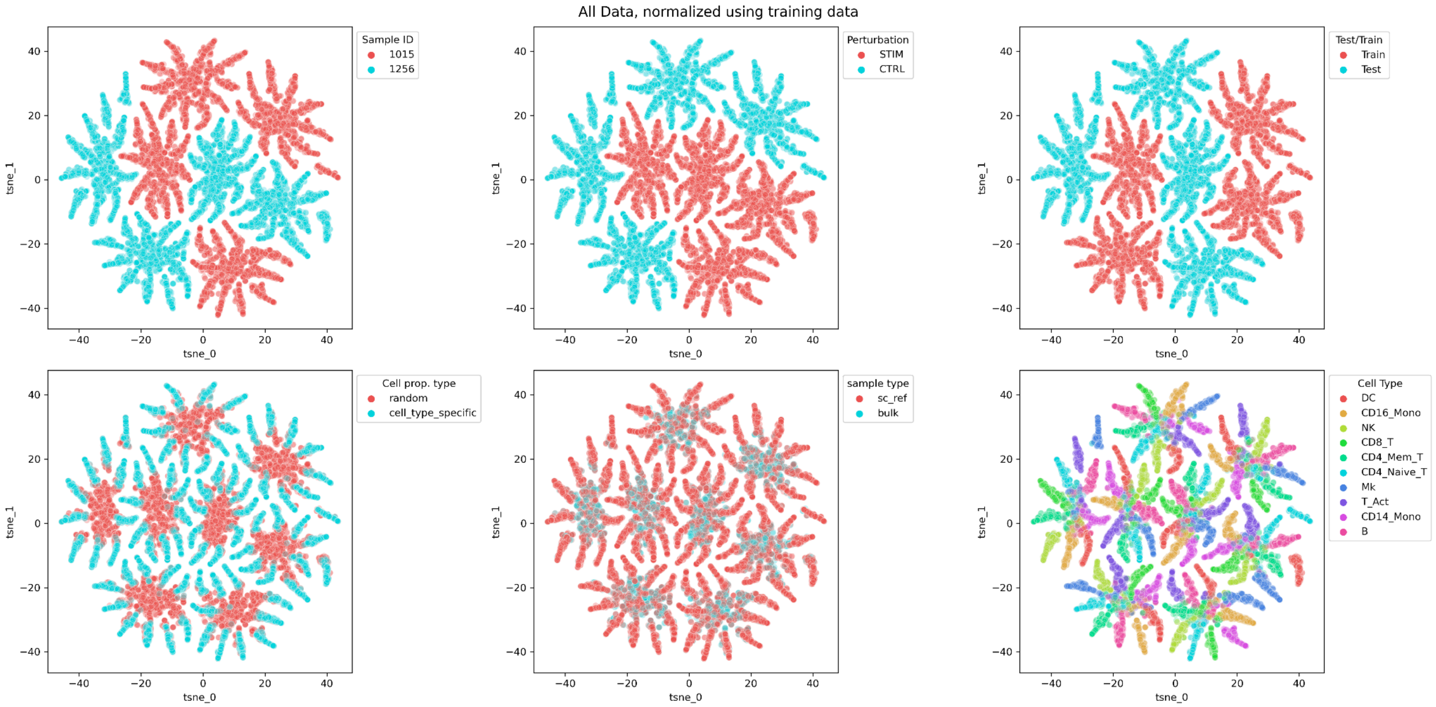

Supp Figure 6. Pseudobulk data generated and colored by source of variation. Our generated data shows independence between, each source of variation, including cell type proportion.
