# Supplementary material for: BuDDI: Bulk Deconvolution with Domain Invariance to predict cell-type-specific perturbations from bulk
Source: PLoS Comput Biol. 2025 Jan 17;21(1):e1012742. doi: 10.1371/journal.pcbi.1012742 (PMC11790236; doi:10.1371/journal.pcbi.1012742)
Supplement: S7 Fig — (PDF) [file pcbi.1012742.s007.pdf]

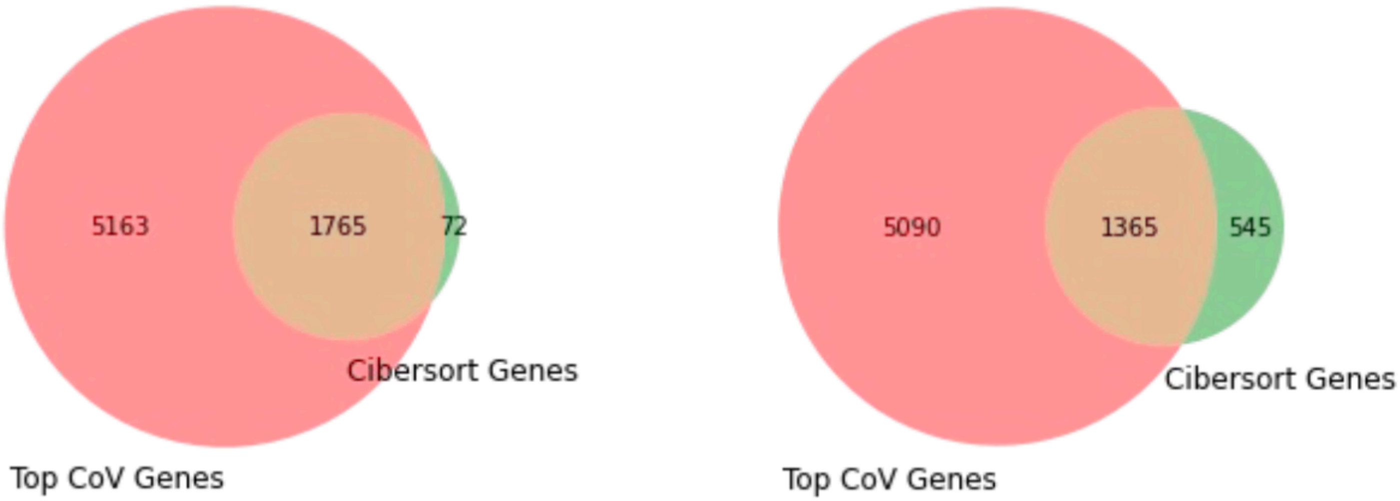

Supp Figure 7. Overlap of top coefficient of variation genes and CIBERSORTx signature genes used in the Kang et al.[1] (left) and sex-dependent liver (right) analyses.

Reference

1. Kang HM, Subramaniam M, Targ S, Nguyen M, Maliskova L, McCarthy E, et al. Multiplexed droplet single-cell RNA-sequencing using natural genetic variation. Nat Biotechnol. 2018;36: 89–94.
