# Supplementary material for: BuDDI: Bulk Deconvolution with Domain Invariance to predict cell-type-specific perturbations from bulk
Source: PLoS Comput Biol. 2025 Jan 17;21(1):e1012742. doi: 10.1371/journal.pcbi.1012742 (PMC11790236; doi:10.1371/journal.pcbi.1012742)
Supplement: S8 Fig — Only samples with sufficient expression were used in our analysis, this includes samples 421, 436, 458, 460, 462, 475, 515, and 542. (PDF) [file pcbi.1012742.s008.pdf]

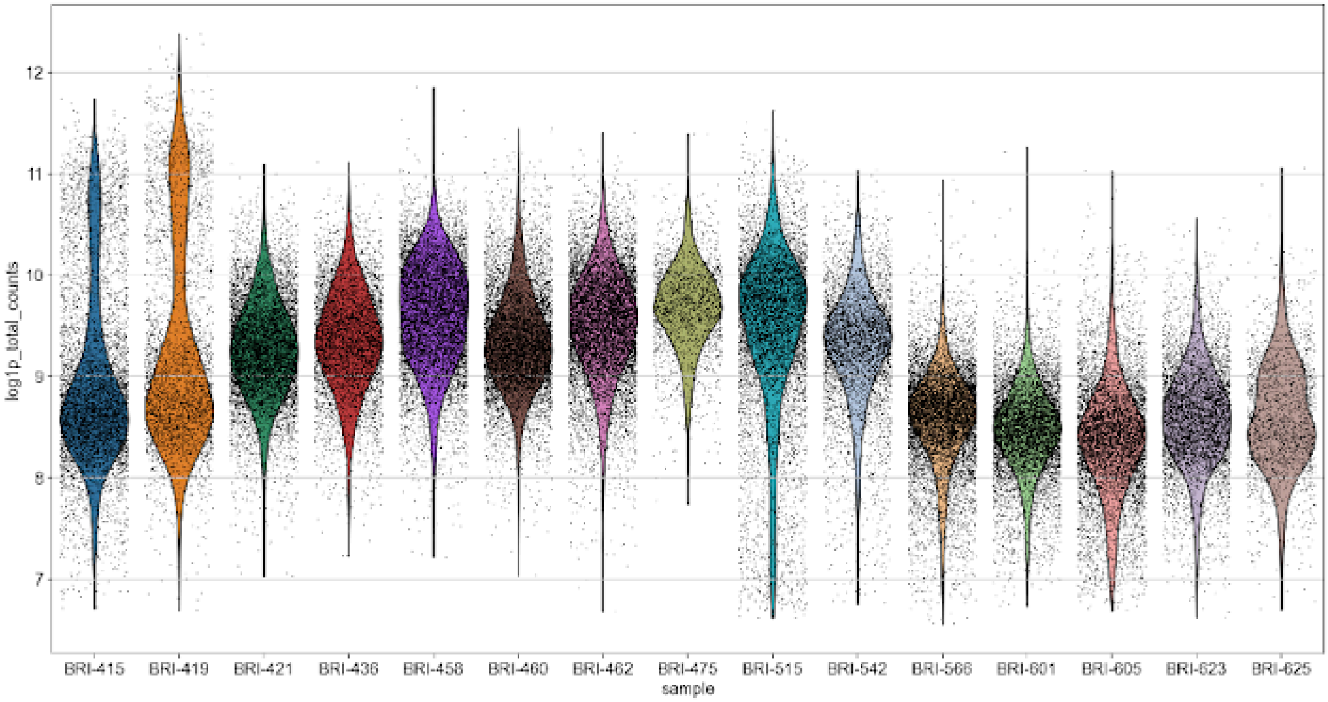

Supp Figure 8. Log total counts for each single-cell synovium sample from Zhang et al.[1]. Only samples with sufficient expression were used in our analysis, this includes samples 421, 436, 458, 460, 462, 475, 515, and 542.

Reference

1. Zhang F, Jonsson AH, Nathan A, Millard N, Curtis M, Xiao Q, et al. Deconstruction of rheumatoid arthritis synovium defines inflammatory subtypes. Nature. 2023;623: 616–624.
