# Supplementary material for: BuDDI: Bulk Deconvolution with Domain Invariance to predict cell-type-specific perturbations from bulk
Source: PLoS Comput Biol. 2025 Jan 17;21(1):e1012742. doi: 10.1371/journal.pcbi.1012742 (PMC11790236; doi:10.1371/journal.pcbi.1012742)
Supplement: S1 Table — (PDF) [file pcbi.1012742.s009.pdf]

Cell Type in Kang et al.	Number of cells after filtering
CD14 Monocyte	4361
CD4 Naive T cells	2504
CD4 Memory T cells	1762
B cells	1363
CD16 Monocytes	1044
CD8 T cells	813
T Activated	633
NK	619
CD	604
Mk	236

Sample ID in Kang et al.	Number of cells after filtering
1015	3177
1256	2396
1499	2280
1244	2031
1016	1484
101	1224
1039	679
107	668

Supp Table 1. Number of cells by cell type and by sample ID in the Kang et al.[1] dataset after filtering.

1. Kang HM, Subramaniam M, Targ S, Nguyen M, Maliskova L, McCarthy E, et al. Multiplexed droplet single-cell RNA-sequencing using natural genetic variation. Nat Biotechnol. 2018;36: 89–94.
