# Supplementary material for: BuDDI: Bulk Deconvolution with Domain Invariance to predict cell-type-specific perturbations from bulk
Source: PLoS Comput Biol. 2025 Jan 17;21(1):e1012742. doi: 10.1371/journal.pcbi.1012742 (PMC11790236; doi:10.1371/journal.pcbi.1012742)
Supplement: S2 Table — (PDF) [file pcbi.1012742.s010.pdf]

cell_ontology_class	B cell	Kupffer cell	NK cell	duct epithelial cell	endothelial cell of hepatic sinusoid	hepatic stellate cell	hepatocyte	myeloid leukocyte	plasmacytoid dendritic cell
**mouse.id**									
**1-M-62**	1	5	2	0	25	2	434	1	1
**1-M-63**	16	609	245	0	495	26	881	34	14
**3-F-56**	0	12	2	0	9	1	363	0	0
**3-F-57**	0	1	0	0	5	0	173	0	0
**3-M-8/9**	0	0	0	2	5	0	453	0	0
**18-F-51**	24	221	109	0	27	2	267	40	8
**21-F-54**	39	25	32	0	14	2	250	4	1
**24-M-58**	0	0	0	0	2	0	74	0	0
**24-M-59**	3	18	7	0	5	1	4	5	0
**30-M-3**	33	1608	56	0	3	0	1	177	7
**30-M-4**	14	3	11	0	5	0	0	12	1
**30-M-5**	73	44	88	0	80	5	29	34	9

Supp Table 2. Number of cells by sample ID and cell type after filtering and before combining the two cell types “endothelial cell of hepatic sinusoid” and “duct epithelial cell”
