# Supplementary material for: BuDDI: Bulk Deconvolution with Domain Invariance to predict cell-type-specific perturbations from bulk
Source: PLoS Comput Biol. 2025 Jan 17;21(1):e1012742. doi: 10.1371/journal.pcbi.1012742 (PMC11790236; doi:10.1371/journal.pcbi.1012742)
Supplement: S3 Table — (PDF) [file pcbi.1012742.s011.pdf]

characteristics: sex	f	m
characteristics: age		
12	2.0	4.0
15	2.0	4.0
18	2.0	4.0
21	2.0	4.0
24	NaN	3.0
27	NaN	4.0
3	2.0	4.0
6	2.0	4.0
9	2.0	4.0

Supp Table 3. Number of bulk liver samples used in analysis by sample ID and age.
