# Supplementary material for: BuDDI: Bulk Deconvolution with Domain Invariance to predict cell-type-specific perturbations from bulk
Source: PLoS Comput Biol. 2025 Jan 17;21(1):e1012742. doi: 10.1371/journal.pcbi.1012742 (PMC11790236; doi:10.1371/journal.pcbi.1012742)
Supplement: S4 Table — (PDF) [file pcbi.1012742.s012.pdf]

cell_type	B	Endothelial	Fibroblast	Myeloid	NK	T_CD4	T_CD8	T_other
sample								
BRI-415	1830	139	855	800	197	2567	703	279
BRI-419	493	242	558	1385	127	715	270	107
BRI-421	427	283	1795	2935	432	2059	772	328
BRI-436	1121	79	68	741	157	1827	1085	577
BRI-458	1322	319	548	4286	59	1791	743	259
BRI-460	811	903	5378	1363	110	297	182	59
BRI-462	771	250	3216	4923	136	858	346	148
BRI-475	87	90	303	941	183	335	263	56
BRI-515	1721	461	1956	546	384	1675	890	311
BRI-542	594	496	293	1175	161	1246	744	271
BRI-566	455	200	2169	299	407	2895	2574	639
BRI-601	253	180	4032	2477	127	834	229	112
BRI-605	2339	558	2083	924	150	1265	412	240
BRI-623	250	317	82	2926	313	991	832	333
BRI-625	80	451	267	595	119	522	193	96

Supp Table 4. Number of cells by sample ID and cell type from Zhang et. al.[1]

1. Zhang F, Jonsson AH, Nathan A, Millard N, Curtis M, Xiao Q, et al. Deconstruction of rheumatoid arthritis synovium defines inflammatory subtypes. Nature. 2023;623: 616–624.
