# Supplementary material for: BuDDI: Bulk Deconvolution with Domain Invariance to predict cell-type-specific perturbations from bulk
Source: PLoS Comput Biol. 2025 Jan 17;21(1):e1012742. doi: 10.1371/journal.pcbi.1012742 (PMC11790236; doi:10.1371/journal.pcbi.1012742)
Supplement: S5 Table — (PDF) [file pcbi.1012742.s013.pdf]

	Range searched	Figure 2	Supp. Figure 2	Figure 3, Full BuDDI model	Figure 4	Figure 5
Learning Rate	0.005	0.005	0.005	0.005	0.005	0.005
Non-slack B	100	100	100	100	100	100
Slack B	0.1	0.1	0.1	0.1	0.1	0.1
Epochs	[50, 100, 200]	200	200	200	200	200
Proportion Classifier weight	[10, 100, 1000, 10000]	100	100	100	100	100
Sample Classifier weight	[10, 100, 1000, 10000]	100	10	100	100	10000
Drug Classifier weight	[10, 100, 1000, 10000]	100	10000	100	10	10000
Technology Classifier	[10, 100, 1000, 10000]	100	10	100	100	100
Batch Size	500	500	500	500	500	500
Hidden Layer 1	512	512	Range searched : [256, 512]<br>Final: 256	512	512	512
Hidden Layer 2	256	256	Range searched : [128, 256]<br>Final: 128	256	256	256
Latent Dimension	64	64	64	64	64	64
Classifier Dimension	64	64	64	64	64	64

Supp Table 5. Hyperparameters for each trained BuDDI model.
