# Supplementary material for: BuDDI: Bulk Deconvolution with Domain Invariance to predict cell-type-specific perturbations from bulk
Source: PLoS Comput Biol. 2025 Jan 17;21(1):e1012742. doi: 10.1371/journal.pcbi.1012742 (PMC11790236; doi:10.1371/journal.pcbi.1012742)
Supplement: Attachment — Submitted filename: PLOS comp bio reviewer response.docx [file pcbi.1012742.s014.docx]

We thank the reviewers for their detailed feedback. We provide the responses to each of the reviewers points below.

Reviewer #1: In this manuscript, Davidson and colleagues present a new computational method to decovolve cell-type perturbations. Their method, BuDDI, uses domain invariance tools to leverage bulk sequencing and single-cell sequencing data to predict cell-type expression shifts during treatment/perturbation.

This is a very well-written manuscript – it is logical and easy to follow. Moreover, I commend the authors for the careful documentation of their code, which they have already posted to github. Overall, I think this tool addresses a pressing need in the field, and I suspect it will be adopted by many labs.

I have only minor comments:
1. The authors use the terminology ‘slack space’ in several portions of the paper. While those with machine learning expertise may understand what the authors intend, I would encourage the authors to a sentence in the results section (during the portion covering Figure 2) to introduce the term more clearly.

We thank the reviewer for reminding us of the audience for the paper. We have now added the following text to the results section introducing the term “slack”:

“Finally, we qualitatively examined whether any variability remained unexplained by the other latent spaces. Specifically, we evaluated the slack latent space, which is designed to capture any residual variability not captured by the supervised or semi-supervised latent spaces.”

2. The discussion is short – I think the manuscript would benefit from more details about how the authors expect BuDDI to be used and expanded. Moreover, the authors mention that BuDDI may be applicable to other data types. I think more speculation here would be welcome. For example, I could certainly see application of BuDDI to methylation or proteomic data. Moreover, do the authors see a future for extending the BuDDI framework to spatial data types?

This is a great point, we have now added the following sentences to the discussion:

“Three natural extensions for BuDDI can be used to unite across 1) single-cell and bulk ATAC-Seq to get cell-type specific peaks, 2) cell-free DNA and ATAC-Seq to detect cell- or tissue-specific fragments, and 3) single-cell RNA-Seq and spatial RNA-Seq to deconvolve spatial spots.”

“The structure of BuDDI is also very modular, easily allowing users to extend BuDDI to include additional contraints, such as a clustering constraint within the sample latent space. This allows the user to identify, for example, tumor subtypes that are independent of confounders captured in other latent spaces.”

3. Finally, it would be helpful to get a sense of how computationally demanding BuDDI is. How well does BuDDI scale to large datasets, and does it compare favorably to BayesPrism/PCA/CVAE/etc…?

We thank the reviewer for their suggestion, we have now added the following line to our manuscript:

“To generate Supp. Fig 2, PCA and CVAE take under four minutes to train and apply to a new dataset, BuDDI takes roughly 25 minutes to train and under 4 for application, BayesPrism takes over two hours for building and applying their model to a subset (<50%) of the entire dataset.”

Reviewer #2: The authors proposed a novel method for integrating single-cell RNA sequencing (scRNA-seq) reference data with corpora of case-control bulk RNA-seq data (named BuDDI) to infer cell-type-specific perturbation effects. BuDDI should help the community bridge this integration gap by learning domain-invariant latent representations within a variational autoencoder (VAE) framework, disentangling sources of variation such as cell type proportion, perturbation effect, and experimental variability. The authors validate BuDDI on simulated and real datasets, demonstrating its ability to predict cell-type-specific perturbation responses and sex-specific differences.

BuDDI addresses the fundamental problem of integrating single-cell and bulk RNA-seq data using domain adaptation techniques and disentangled latent representations within a VAE framework. The results show that BuDDI generally outperforms existing methods in various experimental settings, demonstrating its effectiveness in predicting cell-type-specific responses. Considering the vast amount of existing data, BuDDI has been tested on a few data (i.e., sex-specific differences and drug response in a single context).

In general, the paper is well-written, and the achieved results are promising and well-presented. However, the authors should address some concerns before a possible publication in PLoS Computational Biology.

Major comments
- The authors should consider applying BuDDI to a wider range of real-world datasets, including different tissues, diseases, and perturbations. This would provide a more comprehensive assessment of BuDDI’s generalization abilities and robustness.

We thank the reviewer for their suggestions. We have now applied BuDDI to a sci-Plex3 dataset, a dataset we have not previously analyzed. This dataset contains several perturbations across 3 cell lines that were single-cell sequenced in both perturbed and normal conditions. To evaluate BuDDI, we chose 5 drugs, each with a different method of action or target pathway and computationally created cell-line specific perturbation pseudobulks from a mixture of cell lines. This increases our test comparisons to 15 additional experimental settings (5 drugs by 3 cell-line specific perturbations). Since we created the pseudobulks in a cell-type specific way, we can identify both false positives (i.e. a cell line was predicted to be perturbed by a drug when it was not) and true positives (i.e. identify the true genes that were perturbed by a drug in each cell line). A schematic of the experimental design is provided in Supp. Figure 2. In addition to our supplementary figure, we have added the following text:

“To further validate BuDDI, we assessed its performance across a broader range of cell types and perturbations using sci-Plex3 data (Srivatsan et al. 2020)s. BuDDI was applied to computationally mixed single-cell data from three cell lines (A549, K562, MCF7) and five drugs, each with distinct mechanisms of action. We found that, across all cell lines and drugs, BuDDI consistently demonstrated either superior or comparable false positive rates and area under the PR curve (Supp. Figure 2).”

**Data Processing section:**

The data for the sci-Plex3 analysis were downloaded from NCBI’s GEO (GSM4150378). In this data, we focused on five drugs (Fedratinib, Tanespimycin, Trametinib, Triamcinolone Acetonide, and Trichostatin A) that were previously reported to have an observed response in each of the three cell lines (A549, MCF7, and K562). We removed cells with more than 30% mitochondrial reads and less than 500 genes expressed. Additionally, we kept cells that were treated with no drug or treated at 1000nM. We only kept genes with at least one count in at least 50 cells in each cell line. After filtering, we are left with 13917 cells (MCF7: 7858, K562: 3132, A549: 2927) and 8022 genes across the three cell lines.

**Evaluation of models applied to sci-Plex3 data**

We generated 15 tests to evaluate each model's performance on sci-Plex3 data. Each test corresponds to a single drug and cell line combination. Perturbed pseudobulks were generated from a single perturbed cell line and the two remaining unperturbed cell lines. The non-perturbed pseudobulks were generated from all non-perturbed cell lines. Reference single cell expression was provided only from non-perturbed single cells. Each method was then evaluated for how well it reproduced unseen perturbed cell-type specific expression. All cells were divided into test and training cells, so no cells used in pseudobulk generation were also used for evaluation. True positives were the top differentially expressed genes for each cell line. The metrics used to evaluate how well each model correctly identifies differentially expressed genes were the area under the precision-recall curve for identifying the top 10 or 50 differentially expressed genes. To evaluate the false-positive rate of each method, we identified how many genes were identified as differentially expressed between the non-perturbed cell lines after simulating the cell-line-specific perturbation effect. A gene was identified as differentially expressed if the Bonferroni-corrected p-value was < 0.05 after applying a t-test of ten simulated perturbed and non-perturbed samples. For BayesPrism, a subsample of ten perturbed and non-perturbed samples was used for evaluation. BuDDI, PCA, and CVAE were trained independently three times for evaluation.

- The manuscript could benefit from a more intuitive explanation of the VAE framework and domain adaptation techniques. Additional visualisations or diagrams could help readers understand the model's architecture and how it disentangles sources of variation.

We thank the reviewer for their feedback, we have now added the following statements to ensure the reader has a clearer understanding of VAEs and domain adaptation techniques.

**Introduction**:

“This concept is related to domain invariance, where latent representations are invariant to changes in a domain. For example, if the prediction task is to count the number of cells in an image, and the trained model can do so accurately on images with varying brightness, coloration, and resolution, the model is considered invariant to these differences in the brightness, coloration, and resolution domains.”

**The model structure of BuDDI:**

“We implement BuDDI as a Variational Autoencoder (VAE) (Kingma and Welling 2013), since it is a generative model, straightforward to train, and its modular structure. Briefly, a VAE is a model trained to re-generate the original input data after it has been compressed through a bottleneck layer. The bottleneck layer is typically much smaller than the input data and is reflective of the main sources of variation within the data, which we will refer to as a latent representation of the data. BuDDI extends the VAE structure by having more than one latent representation, and all but one latent representation is directed to capture a specific source of variability - the remaining latent representation, termed the ‘slack’, captures unexplained variability.“

- The authors should benchmark BuDDI against a wider range of existing deconvolution and integration methods. This would provide a more thorough assessment of BuDDI's performance relative to the state-of-the-art.

To our knowledge there is no currently available method that can infer cell-type-specific expression changes from integrating heterogeneous bulk samples across multiple states and non-perturbed single-cell data in a single state. This is why we implemented our own version of PCA+latent projection and CVAE models that can integrate across these two resolutions.

While we include BayesPrism, the current state-of-the-art for cell type deconvolution, deconvolution is not the main goal of BuDDI. Deconvolution methods are currently the closest method available that can unite bulk and single cell in order to infer expression changes, but most utilize the key assumption that bulk and single-cell data come from the same condition. While BayesPrism is not designed to integrate single-cell and bulk data from different conditions, it is the best performing deconvolution method that is able to directly account for differences in variability between single-cell and bulk data. For this reason, we included it in our analyses as a comparator and not other deconvolution methods. We should be clear that this is a use of BayesPrism outside of its designed context, and findings of modest performance don’t indicate that BayesPrism is not good at it’s designated use case, deconvolution.

- Considering the model complexity, BuDDI might overfit especially when dealing with limited real-world data. The authors should discuss the potential for overfitting and present strategies, such as regularisation techniques, to mitigate this issue.

We thank the reviewer for pointing out the need to mitigate overfitting risk. We have added the following text to our discussion:

“The complexity of the model and the amount of available data should be considered by end-users, especially when considering the risk of overfitting when using a limited amount of bulk data. One potential extension of the model could involve incorporating dropout hyperparameters to mitigate this risk. However, it is essential to carefully evaluate held-out data to ensure overfitting does not occur.”

The mathematical derivation of the modified ELBO lacks many steps.

While we provide a sketch of the ELBO derivation for the ease of the reader, this is not our original work and is only augmented from the work we cite (DIVA[^27^](https://paperpile.com/c/ji4U7V/zfFE)). We have now added the following text to allow readers to find the more detailed derivation in the DIVA manuscript.

“A more detailed derivation of $L_{\theta, \phi}(x)$ can be found in the original DIVA manuscript (Ilse et al. 2020).

In addition, the authors should better explain why and how they decided to introduce three classifiers.

We have now added the following text to our discussion:

“Furthermore, while we use three classifiers in most of our evaluations, BuDDI can currently support the use of 2 or 3 classifiers and can be easily extended to add or remove classifiers. In our experiments, three classifiers best represented the independent sources of variation, but this is an experimental design choice that should be made by the end user. ”

Minor comments
- Clarify the definition of "domain invariance": The term "domain invariance" is used throughout the manuscript, but its precise definition is not explicitly stated. A clear definition would help the readers understand the concept and its relevance

We thank the reviewer for pointing this out, we have now provided an example and clearer wording around our usage of “domain invariance” in the introduction.

“This concept is related to domain invariance, where latent representations are invariant to changes in a domain. For example, if the prediction task is to count the number of cells in an image, and the trained model can do so accurately on images with varying brightness, coloration, and resolution, the model is considered invariant to these differences in the brightness, coloration, and resolution domains. One difference between our proposal and typical domain invariance approaches is that our main goal is not for our method to be invariant of unseen domains but invariant to observed domains within our dataset of interest. In our case, we would like to model each latent representation to be independent of one another, which could also be phrased as having latent representations that are disentangled. Specifically, this means that changes in one latent representation are independent of changes in all other latent representations. This framework can be used to learn classifiers invariant to a specific confounding factor24,27 or to analyze the latent spaces to interrogate the sources of variability within the data23,25,26.”

- The authors mention using grid search for hyperparameter tuning (just for some hyperparameters) but do not provide details on the search space or the specific metrics used for optimisation. More information on this process would be helpful for reproducibility. Moreover, the authors decide to manually set other hyperparameters (e.g., learning rate and beta values). How did they select these values? What about the number of layers and neurons per layer?

We added the following text to our methods section to describe our parameter choices: “All model choices that were not searched over were chosen early in model design on simulated data experiments and kept consistent throughout all evaluations.”

We provide the following text regarding our Beta term selection: “The non-slack Beta terms were always set to 100 and x is set to 0.1. This parameter choice encouraged the non-slack latent representations to be biased towards fully capturing the source of variability, since a larger term created a stronger bottleneck on the latent representation and encouraged stronger disentanglement within the latent space56.”

We provide the following text on our learning rate: “the Adam optimizer, with a learning rate of 0.005”

For our grid search, we state: “The number of epochs [50, 100, 200] and the classifier weights [100, 1000, 10000] were identified using grid search of all combinations”

The metric we used for optimization is: “We minimized reconstruction loss and maximized the Spearman correlation of the true and estimated cell type proportions on a training validation set, which is 20% of the training set held out during training.”

We provide the following text for the dimensions of our model: “For all models, excluding the sci-Plex3 model, we used 64 dimensions for each latent representation and a batch size of 500. We used internal dimensions of 512 and 256 for the cell type proportion predictor. We used a single 512-dimensional dense layer for the perturbation and experimental predictors. The full structure of our model, including the dimensions of each hidden layer is provided in **Supp. Fig 5**. All model choices that were not searched over were chosen early in model design on simulated data experiments and kept consistent throughout all evaluations. All hyperparameter choices and ranges searched are provided in **Supp. Table 5.**”

In addition, we now provide all hyperparameter choices and grid search ranges in Supp. Table 5.

- The authors should discuss BuDDI’s limitations, such as its reliance on reference scRNA-seq data and potential challenges in dealing with highly heterogeneous tissues.

We thank the reviewer for pointing this out, we have now extended our discussion to include these points:

“Another caveat to using BuDDI, is that BuDDI assumes that given all sample information, such as cell type proportion, sample ID, perturbation status, sequencing differences etc. a normal pseudobulk and normal bulk should have highly correlated expression. However, this assumption is violated when cell types are missing in single-cell data but present in bulk. While we do learn a technology-specific transformation of the data, this transformation will inherently become entangled with the cell type proportion latent space and degrade our predicted cell-type-specific perturbation responses. The correlated normal pseudobulk and normal bulk assumption is also broken when the reference single-cell data is more variable in expression than a normal pseudobulk. This could be caused by stress induced by cell dissociation. This will only be an issue if the technology-specific transformation is correlated with another modeled source of variability, so if the same stress pathway was activated in the perturbation, our perturbation and technology latent spaces would now be entangled. When it is suspected that the variability between normal pseudobulk and bulks may be inappropriately matched, the user can quantify the level of entanglement by using the SAP score analysis depicted in Supp. Fig 1A to provide guidance on whether the application of BuDDI is appropriate.”

- The authors should also outline potential future directions for the method, such as extending it to other omics or incorporating additional sources of variation.

We thank the reviewer for this comment, we have now added the following text to our discussion:

“Three natural extensions for BuDDI can be used to unite across 1) single-cell and bulk ATAC-Seq to get cell-type specific peaks, 2) cell-free DNA and ATAC-Seq to detect cell- or tissue-specific fragments, and 3) single-cell RNA-Seq and spatial RNA-Seq to deconvolve spatial spots.”

Reviewer #3: The study titled “BuDDI: Bulk Deconvolution with Domain Invariance to predict cell-type-specific perturbations from bulk” by Davidson et al. aims to utilize deep representation learning through variational autoencoders specifically designed to identify components associated with predefined sources of variation enabling the transfer of trained network to single-cell setting for prediction of transcriptional perturbations.

The is an important and non-trivial problem where the availability of bulk sequencing data allows for investigations of across-sample variations whereas single-cell data is generally limited to a handful of samples albeit with increased sensitivity to detect sub-clonal populations of cells.

Overall, I think the authors do a good job of evaluating the proposed model in various settings both qualitatively and quantitatively. Couple minor points remains to be addressed.

- For the perturbation prediction and sampling from the latent space, how is the stochasticity handled? Meaning that for a single pseudo-bulk input sample multiple predictions can be made since sampling from the latent space, are the predictions presented based on mean/median aggregate of multiple samples? This should be clarified in the text.

We thank the reviewer for catching this missing detail. We have now added the following to our methods section: “We also note that by using our sampling and data generation approach, we will generate several examples of a perturbed version of a single, unperturbed sample. To collapse our simulated samples back to a single sample, we aggregate by the median for each gene.”

- The encoder architecture is interesting, and I think should be discussed/contrasted more. Vanilla version of an encoder specifically, given enough capacity, should also capture distinct latent spaces. Effectively a single slack space could capture the sources of variation albeit with different latent dimensions.

We thank the reviewer for pointing out this interesting detail. Yes, this is true, you could get the same effects from a vanilla encoder, and this is part of our motivation for generating Figure 3B, where we show that the slack space will capture the perturbation signal if we did not explicitly model for it. However, this approach does not reflect compositional data generation because the latent space in a vanilla VAE is not disentangled. The main motivation for our structure is that we wanted to have composable latent spaces to generate new data, as well as provide a tool to easily QC our data fitting and visualize if we were capturing the sources of variability appropriately.

To capture this nuanced point, we added the following text to our discussion:

“Furthermore, while we use three classifiers in most of our evaluations, BuDDI can currently support the use of 2 or 3 classifiers and can be extended to add or remove classifiers. It is also possible to remove all classifiers and assign sources of variations to specific nodes in the latent representation post-hoc. However, these subsets of nodes will not necessarily be disentangled, implying that directly changing the value in a single node may affect more than one source of variability. The beta-VAE is a VAE variant that encourages disentangled latent representations but still requires post-hoc data interpretation. BuDDI provides an easy-to-use method to have both transparent and disentangled latent representations. One drawback of BuDDI is that it does not test for the number of relevant sources of variation that best describe the data. In our experiments, three classifiers best represented the independent sources of variation, but this is an experimental design choice that should be made by the end-user”

- Discussion should be expanded including potential limitations of modeling pseudo-bulk from single-cell data to integrate bulk sequencing data with single-cell. Specifically, how would the increased heterogeneity in single-cell data effect model performance and the relevance of pseudo-bulk data to true bulk data.

We thank the reviewer for pointing this out. We have now added the following text discussing caveats of our method to the text:

“Another caveat to using BuDDI, is that BuDDI assumes that given all sample information, such as cell type proportion, sample ID, perturbation status, sequencing differences etc. a normal pseudobulk and normal bulk should have highly correlated expression. However, this assumption is violated when cell types are missing in single-cell data but present in bulk. While we do learn a technology-specific transformation of the data, this transformation will inherently become entangled with the cell type proportion latent space and degrade our predicted cell-type-specific perturbation responses. The correlated normal pseudobulk and normal bulk assumption is also broken when the reference single-cell data is more variable in expression than a normal pseudobulk. This could be caused by stress induced by cell dissociation. This will only be an issue if the technology-specific transformation is correlated with another modeled source of variability, so if the same stress pathway was activated in the perturbation, our perturbation and technology latent spaces would now be entangled. When it is suspected that the variability between normal pseudobulk and bulks may be inappropriately matched, the user can quantify the level of entanglement by using the SAP score analysis depicted in Supp. Figure 1A to provide guidance on whether the application of BuDDI is appropriate.”

- More details should be given regarding the parameter tuning such as batch size, number of layers, latent dimension, β, size etc.

We added the following text to our methods section to describe our parameter choices: “All model choices that were not searched over were chosen early in model design on simulated data experiments and kept consistent throughout all evaluations.”

We provide the following text regarding our Beta term selection: “The non-slack Beta terms were always set to 100 and x is set to 0.1. This parameter choice encouraged the non-slack latent representations to be biased towards fully capturing the source of variability, since a larger term created a stronger bottleneck on the latent representation and encouraged stronger disentanglement within the latent space56.”

We provide the following text on our learning rate: “the Adam optimizer, with a learning rate of 0.005”

For our grid search, we state: “The number of epochs [50, 100, 200] and the classifier weights [100, 1000, 10000] were identified using grid search of all combinations”

The metric we used for optimization is: “We minimized reconstruction loss and maximized the Spearman correlation of the true and estimated cell type proportions on a training validation set, which is 20% of the training set held out during training.”

We provide the following text for the dimensions of our model: “For all models, excluding the sci-Plex3 model, we used 64 dimensions for each latent representation and a batch size of 500. We used internal dimensions of 512 and 256 for the cell type proportion predictor. We used a single 512-dimensional dense layer for the perturbation and experimental predictors. The full structure of our model, including the dimensions of each hidden layer is provided in **Supp. Figure 5**. All model choices that were not searched over were chosen early in model design on simulated data experiments and kept consistent throughout all evaluations. All hyperparameter choices and ranges searched are provided in **Supp. Table 5.**”

In addition, we now provide all hyperparameter choices and grid search ranges in Supp. Table 5.

- Please provide the explained variance ratios in figure 4c.

Thank you for pointing out this inconsistency, we have added the variance explained to the figure now.

- Please specify whether the PCA of latent space corresponding to the cell-type (figure 4c) is showing the held-out female single-cell data.

We thank the reviewer for pointing out this missed detail. To make the figure clearer, we have now additionally colored the points by whether or not they are true bulk samples. The caption now states: “**Panel c** depicts the cell type and sex latent spaces colored by either the most abundant cell type or sex. The data in the PCA plots contains all pseudobulk and real bulk training data.“

- Supp Figure 1, legend should be bold for panels (Panel b,c,d) for uniformity.

Thanks for pointing this out, it has been fixed.
